# Increased hepatic receptor interacting protein kinase 3 expression due to impaired proteasomal functions contributes to alcohol-induced steatosis and liver injury

**DOI:** 10.18632/oncotarget.6893

**Published:** 2016-01-12

**Authors:** Shaogui Wang, Hong-Min Ni, Kenneth Dorko, Sean C. Kumer, Timothy M. Schmitt, Atta Nawabi, Masaaki Komatsu, Heqing Huang, Wen-Xing Ding

**Affiliations:** ^1^ Laboratory of Pharmacology and Toxicology, School of Pharmaceutical Sciences, Sun Yat-sen University, Guangzhou 510006, China; ^2^ Department of Pharmacology, Toxicology and Therapeutics, University of Kansas Medical Center, Kansas City, KS 66160, USA; ^3^ Department of General Surgery, University of Kansas Medical Center, Kansas City, KS 66160, USA; ^4^ Department of Biochemistry, School of Medicine Niigata University, Chuo-ku Niigata 951-8510, Japan

**Keywords:** alcohol, RIP3, steatosis, necroptosis, liver injury

## Abstract

Chronic alcohol exposure increased hepatic receptor-interacting protein kinase (RIP) 3 expression and necroptosis in the liver but its mechanisms are unclear. In the present study, we demonstrated that chronic alcohol feeding plus binge (Gao-binge) increased RIP3 but not RIP1 protein levels in mouse livers. RIP3 knockout mice had decreased serum alanine amino transferase activity and hepatic steatosis but had no effect on hepatic neutrophil infiltration compared with wild type mice after Gao-binge alcohol treatment. The hepatic mRNA levels of RIP3 did not change between Gao-binge and control mice, suggesting that alcohol-induced hepatic RIP3 proteins are regulated at the posttranslational level. We found that Gao-binge treatment decreased the levels of proteasome subunit alpha type-2 (PSMA2) and proteasome 26S subunit, ATPase 1 (PSMC1) and impaired hepatic proteasome function. Pharmacological or genetic inhibition of proteasome resulted in the accumulation of RIP3 in mouse livers. More importantly, human alcoholics had decreased expression of PSMA2 and PSMC1 but increased protein levels of RIP3 compared with healthy human livers. Moreover, pharmacological inhibition of RIP1 decreased Gao-binge-induced hepatic inflammation, neutrophil infiltration and NF-κB subunit (p65) nuclear translocation but failed to protect against steatosis and liver injury induced by Gao-binge alcohol. In conclusion, results from this study suggest that impaired hepatic proteasome function by alcohol exposure may contribute to hepatic accumulation of RIP3 resulting in necroptosis and steatosis while RIP1 kinase activity is important for alcohol-induced inflammation.

## INTRODUCTION

Alcoholic liver disease (ALD) is a major worldwide health problem [1, 2]. Alcohol induces both apoptotic and necrotic cell death, which also contribute to the pathogenesis of ALD. However, the mechanisms regulating alcohol-induced cell death in the case of necrotic cell death are poorly understood.

Accumulating evidence suggests that alcohol induces apoptosis in hepatocytes by activating both intrinsic (mitochondrial) and extrinsic (death receptor regulated) apoptotic pathways [3–5]. Chronic ethanol feeding also depletes mitochondrial glutathione (GSH), increases the expression of CD95, induces the onset of mitochondrial permeability transition, and sensitizes cultured rat hepatocytes to tumor necrosis factor-α (TNF-α)-induced apoptosis [6, 7]. Furthermore, recent evidence also suggests that ethanol-induced endoplasmic reticulum (ER) stress activates interferon regulator factor 3 (IRF3) in order to initialize ethanol-induced hepatocyte apoptosis in mice, which contrasts with the role of IRF3 in innate immunity regulation and inflammation [8].

In addition to apoptosis, cells can also die by necrosis. Necrosis is generally considered as a type of sudden cell death characterized by swollen cell, permeabilized plasma membrane and often accompanied with inflammation *in vivo*. Biochemically, caspase normally is not activated in necrotic cells. However, recent evidence suggests that necrotic cell death can also be tightly regulated, a process termed as necroptosis. Necroptosis is similar in nature to necrosis, but is a caspase-independent programmed form of cell death that depends on receptor interacting protein kinase 1 (RIP1) or 3 (RIP3) [9–14]. TNF-α binds to TNF-α receptor 1 (TNFR1) and triggers the binding of RIP1 with RIP3 through the RIP homotypic interaction motif (RHIM), when cellular caspases are inhibited and inhibitor of apoptosis proteins (cIAPs) are depleted. RIP1 and RIP3 then form an amyloid-like structure termed necrosome, which is stabilized by phosphorylated RIP1 and RIP3. Activated RIP3 then recruits and phosphorylates the mixed lineage kinase domain-like (MLKL) protein to promote its oligomerization and translocation to the plasma membranes resulting in eventual membrane rupture and necrosis [15, 16]. In addition to regulating necrosis, RIP1 also regulates NF-κB activation through cIAP1 and cIAP2-induced polyubiquitination of RIP1. Activated NF-κB upregulates the expression of FLICE-like inhibitory proteins (FLIP) and inhibits caspase-8 activation and apoptosis. Intriguingly, in the absence of cIAPs, RIP1, RIP3, FADD, Caspase-8 and FLIP_L_ form a complex known as ripoptosome which activates caspase-8 to trigger apoptosis independent of pronecrotic kinase activities and MLKL [17–19]. The latter is supported by the observation that while RIP1 kinase-dead knock-in mice are viable, RIP3 kinase-dead D161N knock-in mice die via caspase-8 mediated apoptosis [20]. These findings suggest that both RIP1 and RIP3 can also induce apoptosis in addition to their roles in necroptosis.

Recently, Dr. Nagy's group found that chronic ethanol feeding induced RIP3 expression in mouse livers [21]. Moreover, RIP3 knockout (KO) mice had less liver injury, steatosis, and inflammation compared to control mice after chronic ethanol feeding, implicating the importance of RIP3 in ethanol-induced liver injury and progression of ALD [21]. However, the molecular mechanisms of how ethanol induces hepatic RIP3 expression are not clear. In the present study, we found that chronic alcohol feeding plus binge (Gao-binge) treatment increased RIP3 but not RIP1 protein levels in mouse livers. RIP3 KO mice had decreased serum alanine amino transferase (ALT) activity and steatosis but unchanged neutrophil infiltration compared to that of wild type mice after Gao-binge alcohol treatment. Furthermore, we found that Gao-binge alcohol treatment decreased the levels of proteasome subunit alpha type-2 (PSMA2) and proteasome 26S subunit, ATPase 1 (PSMC1) as well as hepatic proteasome activity. In line with the findings from the animal experiments, we also found decreased expression of PSMA2, PSMC1 and increased protein levels of RIP3 in human alcoholic liver tissues compared to the levels in healthy human livers. Our results thus suggest that impaired hepatic proteasome function by alcohol exposure may contribute to alcohol-induced steatosis and liver injury by inducing necroptosis through blocking proteasomal degradation of RIP3.

## RESULTS

### Induction of RIP3 protein by alcohol/ethanol in mouse livers and primary hepatocytes

**Figure 1:**
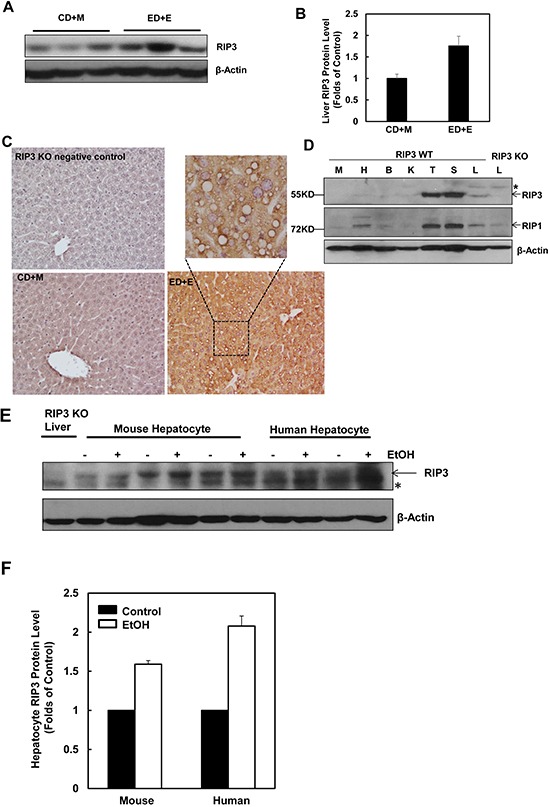
Alcohol treatment induces RIP3 expression in mouse liver, primary mouse and human hepatocytes Male C57BL/6J WT mice were fed ad libitum with the Lieber-DeCarli ethanol liquid diet (10 days,35.5%) followed by a single dose of ethanol (ED+E, 5 g/kg) or pair-fed control liquid diet followed by one dose of maltose dextrin (CD+M, 9 g/kg). Mice were sacrificed 8 hours later after the gavage. **A.** Total liver lysates were subjected to western blot analysis. **B.** Densitometry analysis of (A). The levels of RIP3 were normalized to the loading control (β-Actin). Values represent means±S.E. (*n* = 3 different mice). **C.** Paraffin-embedded liver tissues were subjected to immunohistochemistry for RIP3. Boxed area was enlarged and showed in the top panel. **D.** Total lysates that were extracted from a wild type mouse muscle (M), heart (H), brain (B), kidney (K), thymus (T), spleen (S) and liver (L) were subjected to western blot analysis. The liver lysate from a RIP3 KO mouse was used as a negative control. **E.** Primary cultured mouse and human hepatocytes were treated with ethanol (EtOH, 80 mM) for 6 hours and total cell lysates were subjected to western blot analysis. The liver lysate from a RIP3 KO mouse was used as a negative control. **F.** Densitometry analysis of (E). The levels of RIP3 were normalized to the loading control (β-Actin). Values represent means±S.E. (*n* = 3 independent experiments).

To determine the changes of hepatic RIP3 in the Gao-binge alcohol model, total liver lysates from mice that were fed with ethanol diet followed by ethanol binge (ED+E) or pair-fed control liquid diet followed by maltose dextrin binge (CD+M) were subjected to western blot analysis. We found that the levels of hepatic RIP3 increased almost 2-fold in Gao-binge alcohol-treated mouse livers compare to the control mouse livers (Figure 1A & 1B). Immunohistochemistry analysis of liver tissues also revealed a marked induction of RIP3 in Gao-binge alcohol-treated mouse livers compared to control (CD+M)-treated mouse livers. RIP3 staining increased robustly in hepatocytes and displayed speckles in the cytosol after Gao-binge alcohol treatment, which may reflect the formation of necrosomes (Figure 1C). These results are in general agreement with a previous study that showed increased RIP3 induction in mice that were fed with an ethanol-containing diet for 25 days [22]. We next determined the expression levels of RIP3 and RIP1 in different mouse tissues. We found that both RIP3 and RIP1 were predominantly expressed in mouse thymus and spleen but were almost undetectable in other tissues including muscle, heart, kidney and brain. Both RIP3 and RIP1 proteins were detected in the liver although the expression levels were much lower compared with the thymus and spleen. The lack of 55KD signal in the extract from the RIP3 knockout mouse liver suggests that the antibody that we used for RIP3 is specific (Figure 1D). To determine whether ethanol could affect the expression of RIP3 directly in hepatocytes, primary cultured mouse and human hepatocytes were treated with ethanol (80 mM) for 6 hours. Under these conditions, we have previously demonstrated that ethanol induces expression of many autophagy-related genes [32]. Interestingly, we also found that ethanol treatment increased RIP3 protein levels in both mouse and human primary hepatocytes (Figure 1E & 1F). Taken together, these results indicate that hepatic RIP3 proteins are induced by alcohol in mice that were exposed to a clinical relevant chronic plus binge condition. Ethanol directly induces RIP3 protein in mouse and human hepatocytes independent of other cell types.

### RIP3 knockout mice have less liver injury and steatosis in response to Gao-binge alcohol treatment

**Figure 2:**
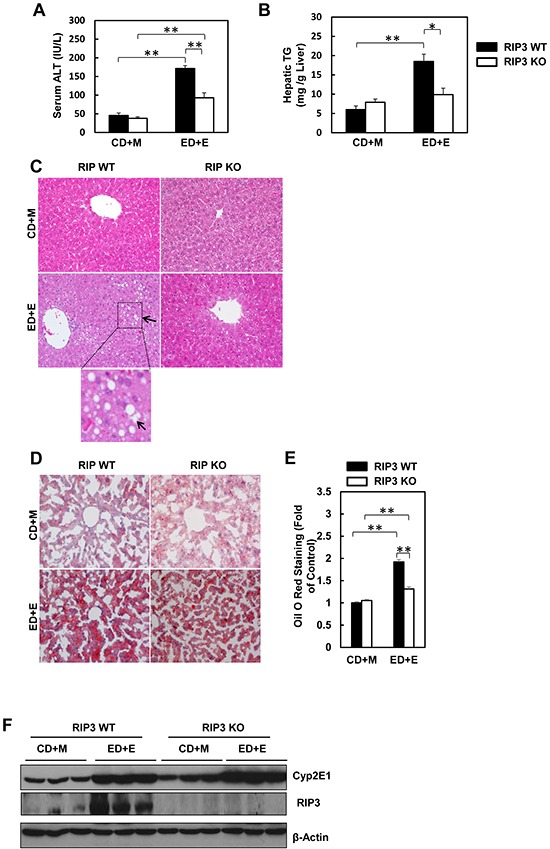
RIP3 KO mice are resistant to Gao-binge alcohol-induced liver injury and steatosis Serum ALT **A.** and liver triglyceride levels **B.** were measured from WT and RIP3 KO mice after Gao-binge treatment. Data shown are means ± S.E. (*n* = 3-4 mice per group. *p<0.05; ** p<0.01 by one-way ANOVA). **C.** Representative H&E images from the WT and RIP3 KO mice after Gao-binge treatment. Lower panel is a photograph from the boxed area. Arrow denotes lipid droplets. **D.** Representative images are shown for Oil Red O staining for the Gao-binge treatment (200x magnification) and Oil Red O staining was quantified using ImageJ software **E.** At least 6 images from each mouse were quantified and data were normalized to the control group. Data shown are means ± S.E. (*n* = 3 mice per group. *p<0.05; ** p<0.01 by one-way ANOVA). **F.** Total liver lysates were analyzed by western blot analysis (*n* = 3 different mice).

We found that the serum ALT levels were significantly elevated in Gao-binge-treated wild type mice, while such an elevation was diminished in RIP3 KO mice (Figure 2A). Moreover, Gao-binge alcohol treatment also significantly increased hepatic triglyceride levels in wild type mice while this increase was inhibited in RIP3 KO mice (Figure 2B). Histological analysis by H & E staining also revealed increased steatosis as demonstrated by increased lipid vacuoles in Gao-binge alcohol-treated wild type mice (arrows, Figure 2C), which was markedly attenuated in RIP3 KO mice. These findings were further confirmed by diminished Oil Red O staining for hepatic lipids in Gao-binge alcohol-treated RIP3 KO mouse livers compared to wild type mouse livers (Figure 2D & 2E). It is known that alcohol consumption can induce hepatic cytochrome P450 2E1 (CYP2E1) expression. Indeed, we also found that Gao-binge alcohol treatment markedly increased the expression of hepatic CYP2E1 in both wild type and RIP3 KO mice. These data suggest that ethanol induces CYP2E1 independent of RIP3. While Gao-binge alcohol treatment increased the protein levels of RIP3 in the mouse livers, no RIP3 expression was detected in the RIP3 KO mouse livers (Figure 2F), confirming again that the antibody that we used for RIP3 is specific for mouse RIP3. Collectively, these results indicate that RIP3 KO mice are more resistant to Gao-binge alcohol treatment-induced liver injury and steatosis compared to wild type mice.

### Changes of hepatic inflammation after Gao-binge alcohol treatment in wild type and RIP3 knockout mice

**Figure 3:**
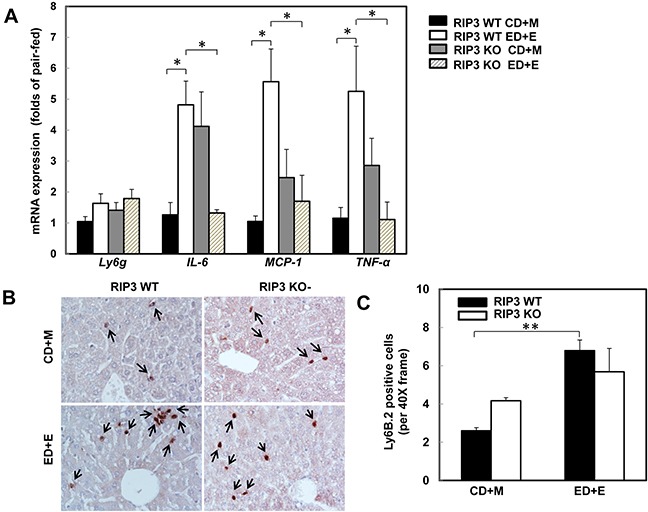
RIP3 KO mice decrease the hepatic expression of inflammatory cytokine genes but have no effects on Gao-binge alcohol-induced neutrophil infiltration WT and RIP3 KO mice were treated with the Gao-binge model. **A.** RNA from mouse livers was used to measure gene expression by qPCR. Results were normalized to β-actin and expressed as fold change compared to WT control. Data shown are means ± S.E (*n* = 3-4 mice per group. *p<0.05; by one-way ANOVA). **B.** Representative images were shown from Ly6B immunohistochemistry after Gao-binge treatment. Arrows denote the Ly6B positive neutrophils. **C.** The number of Ly6B positive neutrophils was counted from at least 22 areas (400 X magnification) each mouse. Results shown are means ± S.E. (*n* = 4-6 mice per group. **p<0.01 compared to WT control by one-way ANOVA).

One of the important features of alcoholic liver disease is increased hepatic inflammation, which can be found in the mouse livers after Gao-binge alcohol treatment [1, 25, 33]. Since necrotic cell death often leads to inflammation, we next determined the hepatic expression of several inflammatory genes in wild type and RIP3 KO mice after Gao-binge treatment. Gao-binge alcohol treatment increased the hepatic expression of *IL-6, MCP-1* and *TNF-α* but not *Ly6g* in wild type mice, which was significantly blunted in RIP3 KO mice despite the basal hepatic expression levels of *IL-6*, *MCP-1* and *TNF-α* were higher in RIP3 KO mice compared to wild type control mice (Figure 3A). We found that Gao-binge alcohol treatment increased the number of hepatic neutrophils by almost 3-fold compared to control treatment but the numbers of hepatic neutrophils between wild type and RIP3 KO mice were similar (Figure 3B & 3C). These data suggest that Gao-binge alcohol treatment may cause mild hepatic inflammation. While the lack of RIP3 seems to affect the expression of some inflammatory genes (*IL-6, MCP-1* and *TNF-α*) at the basal level and after Gao-binge alcohol treatment, the neutrophil infiltration is likely not associated with RIP3.

### 7-Cl-O-Nec-1 treatment attenuates Gao-binge alcohol-mediated inflammation but not liver injury

**Figure 4:**
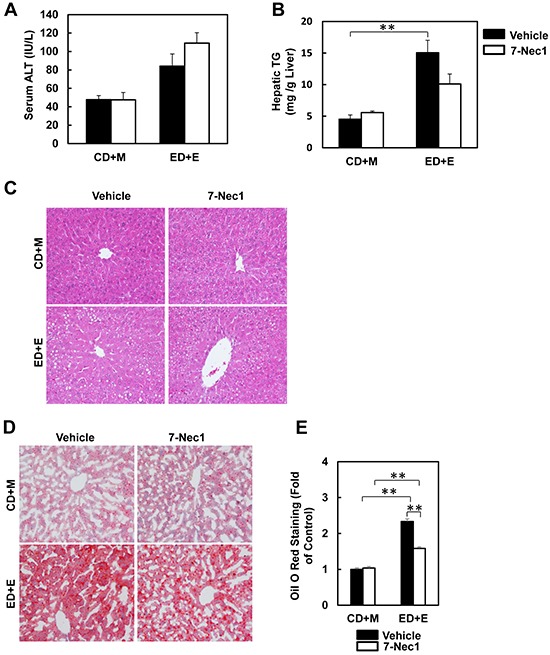
7-Nec1 does not protect against Gao-binge alcohol treatment-induced liver injury and steatosis WT mice were treated with Gao-binge model. At the last day of feeding, mice were either injected with 7-Nec1 (i.p., 5 mg/kg) or vehicle (4% methy-β-cyclodextran in PBS, pH7.4) before ethanol (5 g/kg) or maltose dextrin (9 g/kg) gavage. **A.** Serum ALT levels were determined and **B.** hepatic triglyceride contents were measured using whole liver homogenates. Data shown are means ± S.E. (*n* = 4-6 mice per group. *p<0.05; ** p<0.01 by one-way ANOVA). **C.** Representative H&E images and **D.** Oil Red O staining images are shown (200x magnification). Oil Red O staining was quantified using ImageJ software **E.** At least 6 images from each mouse were quantified and data were normalized to the control group. Data shown are means ± S.E. (*n* = 3 mice per group. *p<0.05; ** p<0.01 by one-way ANOVA).

**Figure 5:**
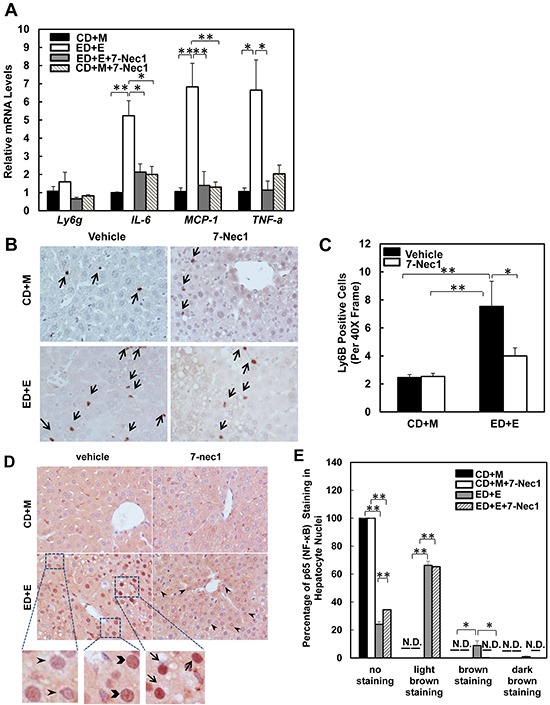
7-Nec1 decreases hepatic inflammation induced by Gao-binge alcohol treatment **A.** RNA from mouse livers was used to measure gene expression by qPCR. Results were normalized to β-actin and expressed as fold change compared to WT control. Data shown are means ± S.E (*n* = 4-5 mice per group. *p<0.05; **p<0.01 by one-way ANOVA). **B.** Representative images are shown from Ly6B immunohistochemistry. Arrows denote the Ly6B positive neutrophils. **C.** The number of Ly6B positive neutrophils was counted from at least 20 areas (400 X magnification) each mouse. Results shown are means ± S.E. (*n* = 3-5 mice per group. *p<0.05 compared to WT control by one-way ANOVA). **D.** Paraffin-embedded liver tissues were subjected to immunohistochemistry for NF-κB subunit p65. Representative images were shown after Gao-binge alcohol treatment with or without 7-nec1. Boxed areas were enlarged and shown in the lower panel. The arrowheads represent light brown staining of p65, the chevrons show brown staining of p65 and the arrows indicate dark brown staining of p65 in hepatocyte nuclei. **E.** The percentage of different intensities of p65 staining. Results are presented as means± S.E. (*n* = 3-4, *p<0.05; **p<0.01 by one-way ANOVA). N.D., not detected.

7-Cl-O-Nec-1 (7-Nec1) is a more potent and specific RIP1 inhibitor, which can suppress RIP1 kinase activity without affecting IDO compared to the old generation of RIP1 kinase inhibitor Necrostatin 1 [34]. If ethanol-induced liver injury is also dependent on RIP1 in addition to RIP3, 7-Nec1 should be able to inhibit liver injury induced by alcohol. To test this hypothesis, the ethanol fed mice were treated with 7-Nec1 before giving a binge of ethanol at the last day of feeding. 7-Nec1 administration did not attenuate ethanol-induced elevation of serum ALT levels (Figure 4A). While the levels of hepatic TG induced by Gao-bine alcohol treatment were reduced by 7-Nec1 by approximately 30%, this reduction did not reach statistical significance (Figure 4B). Histological analysis by H & E staining (Figure 4C) and Oil Red O staining (Figure 4D & 4E) for hepatic lipids showed significantly decreased steatosis in 7-Nec1-treated mice compared to the Gao-binge-treated mice. Moreover, increased expressions of inflammatory genes (*IL-6, MCP-1* and *TNF-α*) by Gao-binge alcohol treatment were also significantly blunted by 7-Nec1 treatment (Figure 5A). Similarly, increased hepatic neutrophil infiltration by Gao-binge alcohol treatment was also markedly suppressed by 7-Nec1 treatment (Figure 5B & 5C). The number of cells with nuclear staining for NF-κB subunit p65 (brown staining), a marker to indicate the activation of NF-kB, was increased by Gao-binge treatment but this increase was significantly suppressed by 7-Nec1 treatment (Figure 5D & 5E). These results suggest that RIP1 Kinase may not be essential for Gao-binge alcohol-induced necropotosis but may promote alcohol-induced hepatic steatosis and inflammation.

### Gao-binge alcohol treatment decreases hepatic RIP1 expression in mice

**Figure 6:**
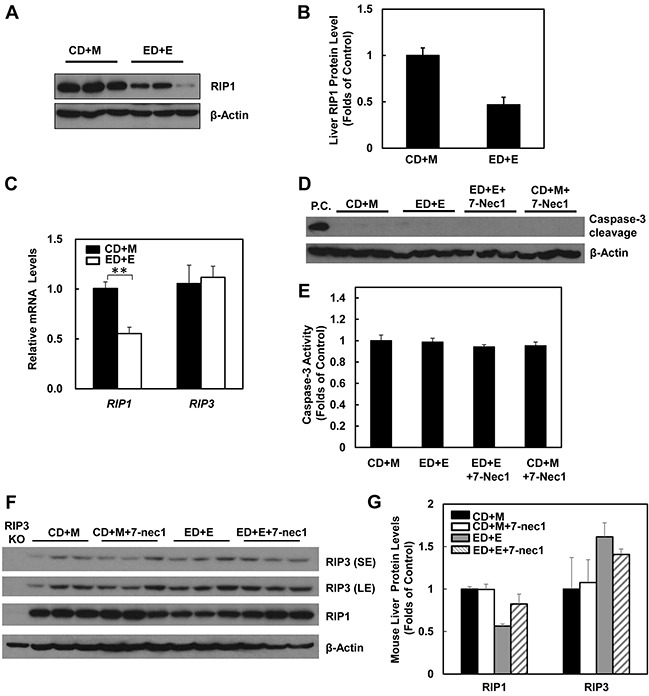
Gao-binge treatment decreases RIP1 expression and 7-Nec1 treatment does not affect RIP1 and RIP3 expression as well as caspase-3 activation WT mice were treated with Gao-binge model. **A.** Total liver lysates were subjected to western blot analysis. **B.** Densitometry analysis of (A). The levels of RIP1 were normalized to the loading control (β-Actin). Values represent means±S.E. (*n* = 3). **C.** RNA from mouse livers was used to measure gene expression by qPCR. Results were normalized to β-actin and expressed as fold change compared to WT control. Data shown are means ± S.E (*n* = 3-4 mice per group. *p<0.05 by one-way ANOVA). **D.** Total liver lysates were subjected to western blot analysis (an LPS/GalN-treated mouse liver lysate was used as a positive control (P.C.) for cleaved caspse-3). **E.** Caspase-3 activities were measured using total liver lysates. Values represent means±S.E. (*n* = 3, no statistical significant difference). **F.** Total liver lysates were subjected to western blot analysis. **G.** Densitometry analysis of (F) (*n* = 3 mice, a RIP3 KO mouse liver lysate was used as a negative control).

**Figure 7:**
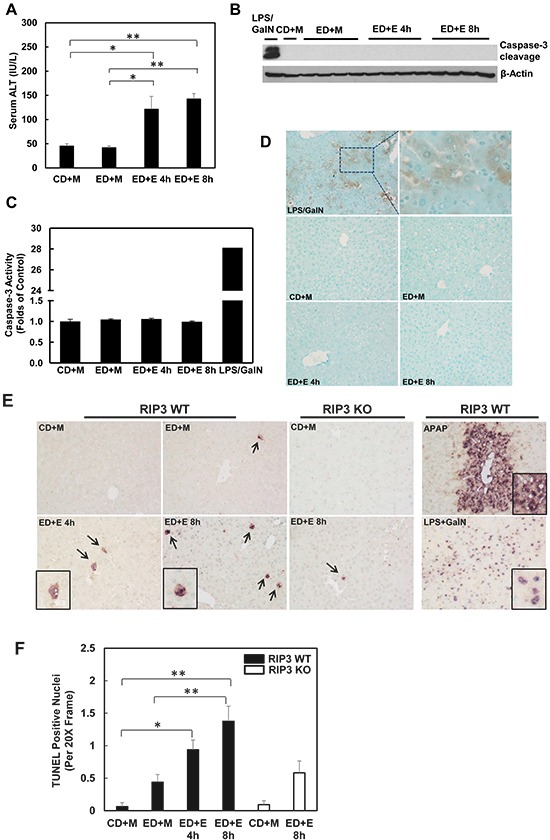
Gao-binge treatment induces necrosis but not apoptosis in mouse livers WT mice were fed with control or ethanol diet for 10 days and at day 10 the mice were either gavaged with maltose or ethanol for 4 or 8 hours. **A.** Serum ALT levels were determined. (*n* = 4, *p<0.05, **p<0.01 by one-way ANOVA). **B.** Total liver lysates were subjected to western blot analysis (an LPS/GalN-treated mouse liver lysate was used as a positive control for cleaved caspase-3). **C.** Caspase-3 activities were measured using total liver lysates. Values represent means±S.E. (*n* = 4, no statistical significant difference). **D.** Representative images of immunohistochemistry for cleaved caspase-3. A mouse liver from LPS/GalN treatment served as a positive control. **E.** Paraffin-embedded liver tissues were subjected to immunohistochemistry for TUNEL staining. APAP group and LPS/GalN group served as positive controls for necrosis and apoptosis, respectively. **F.** The numbers of TUENL positive nuclei were counted from at least 8 areas (200 X magnification) each mouse. Results shown are means ± S.E. (*n* = 3-5 mice per group. *p<0.05, **p<0.01 compared to WT control by one-way ANOVA).

The lack of protection of 7-Nec1 against Gao-binge alcohol treatment-induced liver injury led us to wonder whether Gao-binge alcohol treatment would affect hepatic RIP1 levels. In contrast to RIP3, we found that the protein levels of hepatic RIP1 were markedly decreased in Gao-binge alcohol-treated mouse livers compare to the control mouse livers (Figure 6A & 6B). Moreover, we found that Gao-binge alcohol treatment significantly decreased the mRNA levels of hepatic RIP1 while did not affect the expression of hepatic RIP3 gene (Figure 6C). These data suggest that the levels of hepatic RIP1 and RIP3 are differentially regulated by alcohol. Alcohol may regulate hepatic RIP1 at the transcriptional level but may largely regulate hepatic RIP3 at the posttranslational level. To determine whether inhibition of RIP1 by 7-Nec1 may switch cell death to apoptosis, we determined caspase-3 activation after Gao-binge alcohol treatment in the absence and presence of 7-Nec1. We did not detect cleaved caspase-3 forms in all the treatment groups (Figure 6D). The caspase-3 activities remained unchanged either after Gao-binge alcohol treatment or after Gao-binge alcohol with 7-Nec1 treatment (Figure 6E). Moreover, 7-Nec1 treatment did not affect the hepatic RIP3 levels regardless of the alcohol treatment although it slightly increased RIP1 levels after Gao-binge treatment (Figure 6F & 6G). To determine whether caspase-3 and apoptosis could be activated in the early time points, we also determined the caspase-3 activation after the chronic feeding and gavaged with either maltose or ethanol for 4 hours. We found that ethanol feeding with maltose gavage (ED+M) did not alter the serum ALT levels, but ethanol feeding with one single ethanol gavage (ED+E) for 4 or 8 hours significantly increased serum ALT levels (Figure 7A). However, no changes on caspase-3 activities or caspase-3 cleavage were detected either by western blot analysis or immunostaining after Gao-binge treatment (Figure 7B–7D). TUNEL staining is generally used to detect cells with DNA breakage/fragmentation and can also be used to differentiate apoptosis vs necrosis in hepatocytes [35]. As can be seen, in acetaminophen (APAP)-induced necrosis, TUNEL staining displayed a diffuse pattern in hepatocytes due to the leakage of the fragmented DNA into the cytosol from the nuclei (Figure 7E). In contrast, in LPS/GalN-induced apoptosis, TUNEL staining mainly occurred in the nuclei. We found that TUNEL staining mainly showed a diffuse pattern in Gao-binge alcohol-treated mouse livers, which is very similar to APAP-induced necrosis (Figure 7E arrows). The number of necrotic cells increased at 4 and 8 hours after ethanol binge, which was reduced more than 60% in RIP3 KO mice compared to WT mice although it did not reach statistic difference (Figure 7F). These data suggest that Gao-binge alcohol treatment predominantly induces necrosis but not apoptosis in mouse livers. Inhibition of RIP1 by 7-Nec1 does not induce caspase-3 mediated-apoptosis in the mouse liver.

### Alcohol treatment decreases hepatic proteasomal function

**Figure 8:**
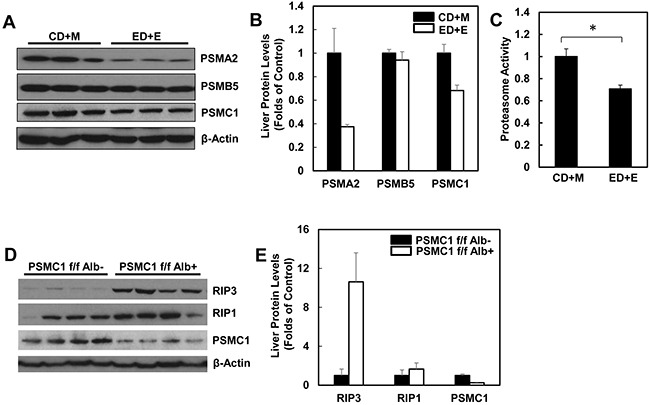
Alcohol impairs hepatic proteasome function and genetic inhibition of proteasome increases protein levels of hepatic RIP1 and RIP3 WT mice were treated with Gao-binge model. **A.** Total liver lysates were subjected to western blot analysis. **B.** Densitometry analysis of (A). The levels of PSMA2, PSMC1 and PSMB5 were normalized to the loading control (β-Actin). Values represent means±S.E. (*n* = 3). **C.** Twenty micrograms of total liver lysates were used to measure the 20S proteasomal activities using a fluorogenic substrate. Results are presented as means± S.E. (*n* = 3, *p<0.05 by Student t test). **D.** Total liver lysates from PSMC1 flox/flox, albumin Cre negative (PSMC1 f/f) and PSMC1 flox/flox, albumin Cre positive (PSMC1 f/f Alb) were subjected to western blot analysis. **E.** Densitometry analysis of (D). Values represent means±S.E. (*n* = 3).

**Figure 9:**
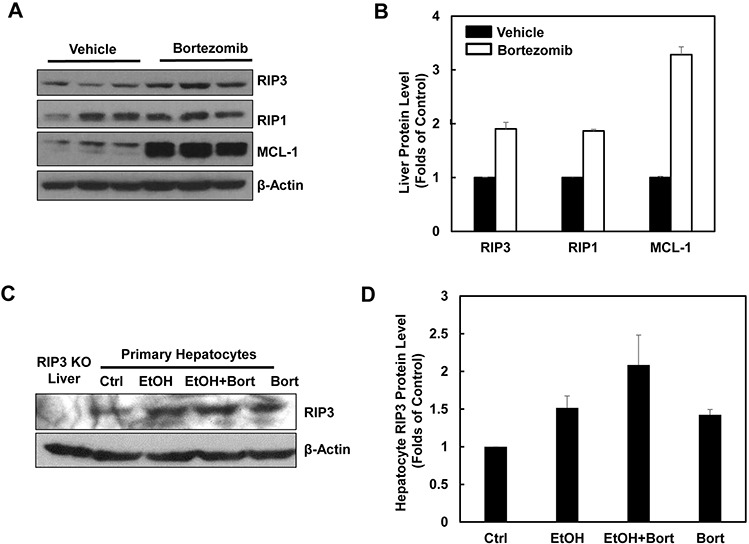
Pharmacological inhibition of proteasome increases protein levels of hepatic RIP1 and RIP3 **A.** WT mice were either injected with Bortezomib (i.p., 1 mg/kg) or vehicle (0.02% DMSO in PBS) for 24 hours followed by a second booster injection for another 6 hours. Total liver lysates were subjected to western blot analysis. **B.** Densitometry analysis of (A). The levels of RIP3, RIP1 and MCL-1 were normalized to the loading control (β-Actin). Values represent means±S.E. (*n* = 3). **C.** Primary cultured mouse hepatocytes were treated with ethanol (80 mM) in the presence or absence of Bortezomib (200 nM) for 6 hours. Total cell lysates were subjected to western blot analysis. A liver lysate from RIP3 KO mouse liver was used as a negative control. **D.** Densitometry analysis of (C). Values represent means±S.E. (*n* = 3 independent experiments).

Since we found that alcohol treatment did not alter the hepatic mRNA levels of RIP3, we hypothesized that alcohol-induced hepatic RIP3 proteins may be regulated at the posttranslational level. We first determined the changes of hepatic proteasome after alcohol treatment since both RIPK1 and RIPK3 are known to be ubiquitinated by the E3 ligases cIAP1 and cIAP2 [36]. We found that the hepatic protein levels of PSMA2 and PSMC1 but not PSMB5 decreased after Gao-binge alcohol treatment compared to the control group mice (Figure 8A & 8B). As a result, Gao-binge alcohol treatment significantly decreased hepatic proteasomal activities compared to the control group mice (Figure 8C). Furthermore, the protein level of RIP3 markedly increased (almost 10 fold) in liver-specific PSMC1 KO mouse livers compared with the matched control WT mice. The protein level of RIP1 also increased in liver-specific PSMC1 KO mouse livers although to a less extent compared to the levels of RIP3 (Figure 8D & 8E). The marked reduction of PSMC1 protein levels in liver-specific PSMC1 KO mouse livers compared with the matched control WT mice indicated the successful deletion of PSMC1 in mouse livers (Figure 8D & 8E). To further determine whether blocking proteasomal functions could affect RIP1 and RIP3 proteins in hepatocytes, we treated mice with Bortezomib, a potent and specific proteasome inhibitor. We found that Bortezomib treatment increased both RIP1 and RIP3 protein levels in the mouse livers (Figure 9A & 9B). Myeloid Cell Leukemia **1** (MCL-1) is an anti-apoptotic Bcl-2 family protein whose level is known to be regulated by the proteasome [37, 38]. We found that Bortezomib treatment also markedly increased hepatic MCL-1 levels, suggesting that Bortezomib inhibits proteasomal functions effectively in the mouse liver (Figure 9A & 9B). In line with these *in vivo* findings, we found that ethanol or Bortezomib treatment increased RIP3 protein levels in primary cultured mouse hepatocytes. Ethanol plus Bortezomib treatment together further enhanced RIP3 protein levels slightly (Figure 9C & 9D). These data indicate that increased hepatic RIP3 protein by alcohol is likely due to the impaired hepatic proteasomal functions induced by alcohol.

### Impaired proteasomal functions and increased hepatic RIP3 protein levels in human ALD

**Figure 10:**
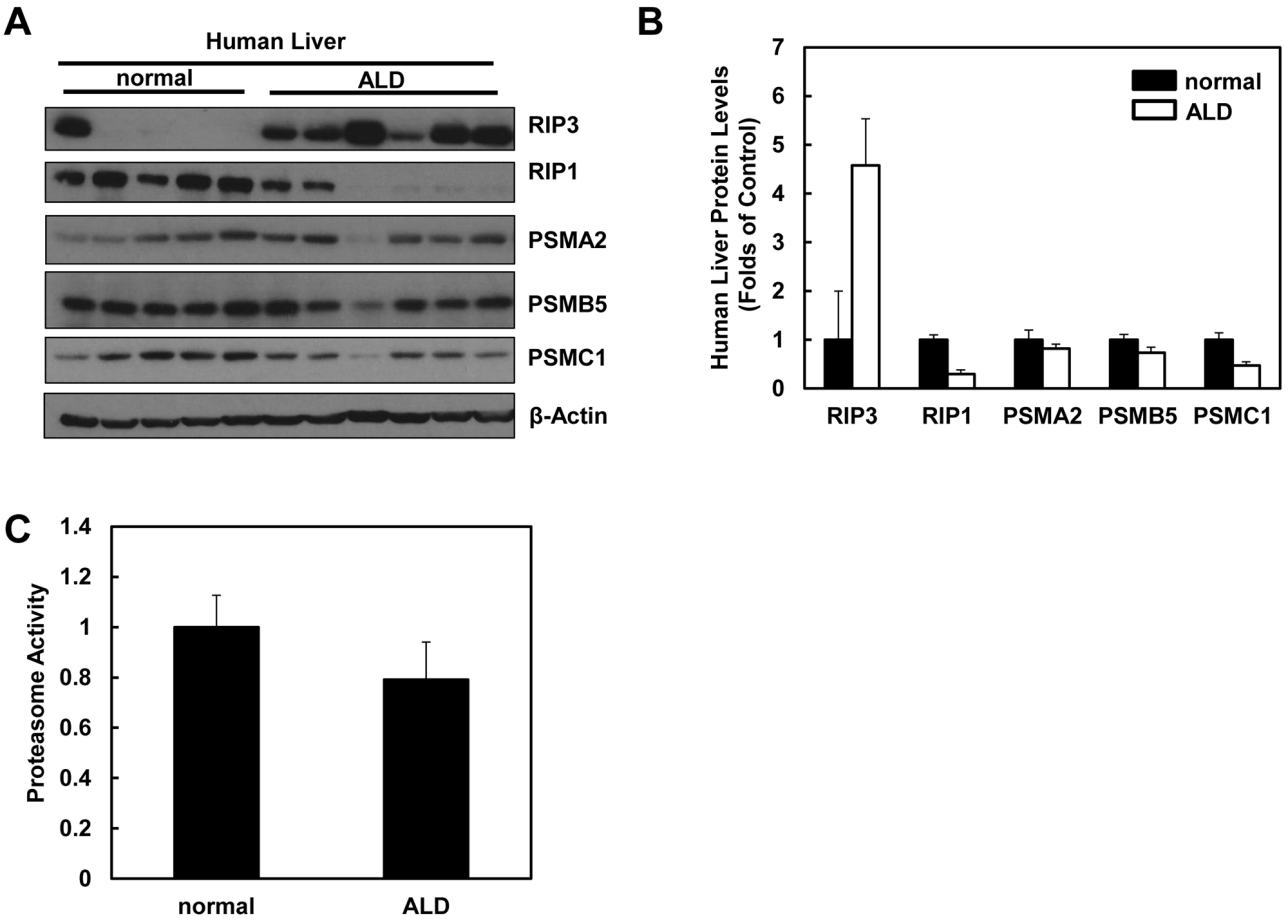
Human ALD livers have altered protein levels of RIP1, RIP3 and proteasome subunit proteins compared to healthy human livers **A.** Total liver lysates from normal human liver and human ALD were subjected to western blot analysis. **B.** Densitometry analysis of (A). The levels of each protein were normalized to the loading control (β-Actin). Values represent means±S.E. (*n* = 5-6). **C.** Twenty micrograms of total liver lysates were used to measure the 20S proteasomal activities using a fluorogenic substrate. Results are presented as means± S.E. (*n* = 5, *p<0.05 by Student t test).

We next determined the hepatic RIP3 protein levels and proteasomal functions in liver samples obtained from human ALD and normal liver tissues. In agreement with our findings from Gao-binge alcohol-treated mouse livers, we found that the hepatic RIP3 protein levels markedly increased whereas RIP1 protein levels decreased in human ALD livers compared to normal human livers (Figure 10A & 10B). Furthermore, we also found that the protein levels of PSMA2, PSMC1 and PSMB5 decreased slightly in human ALD livers compared to normal human livers (Figure 10A & 10B). Similarly, hepatic proteasomal activities also decreased in human ALD livers compared to the normal human livers although this decrease did not reach statistical difference (Figure 10C). These results suggest that the impaired proteasomal function and increased hepatic RIP3 by alcohol exposure in mice may also occur in human ALD.

## DISCUSSION

Liver cell death including apoptosis and necrosis has been documented in experimental and human ALD, and is associated with the progression of ALD. In a chronic alcohol feeding mouse model, inhibition of apoptosis either by genetic deletion of Bid (a BH3-only pro-apoptotic protein) or using a pan-caspase inhibitor VX166, did not protect against alcohol-induced liver injury and steatosis [39]. These data suggest that other forms of cell death are also involved in the pathogenesis of ALD in addition to apoptosis. Here, using a recent established chronic plus binge alcohol model (Gao-binge model), we showed that RIP3 but not RIP1 was induced in mouse livers. Moreover, serum ALT activity and hepatic steatosis but not hepatic neutrophil infiltration was reduced in RIP3 KO mice compared to wild type mice. Furthermore, the protein levels of PSMA2 and PSMC1 as well as hepatic proteasome activity decreased in Gao-binge alcohol-treated mouse livers. More importantly, we also found decreased expression of PSMA2 and PSMC1 but increased protein levels of RIP3 in human alcoholic liver tissues compared to the levels in healthy human livers. These results suggest that impaired hepatic proteasome function by alcohol exposure may lead to the stabilization of RIP3 proteins and contribute to alcohol-induced steatosis and liver injury.

It is well known that alcohol consumption increases the gut permeability resulting in increased levels of hepatic portal lipopolysaccharides (LPS) and production of TNF-α in the liver [40, 41]. TNF receptor 1 (TNFR1) KO mice are resistant to alcohol-induced liver injury and steatosis, underlying the critical role of TNF-α in the pathogenesis of ALD [42]. TNF-α is a pleiotropic cytokine with multiple functions in inflammation, cell survival and cell death. For cell death, depending on the cellular context, TNF-α can induce caspase-dependent apoptosis and RIP-mediated necroptosis. Although there are two TNF receptors that have been identified, the diverse effects of TNF-α mainly depends on TNFR1. Upon binding with TNFR1, TNF-α triggers the formation of the complex including TRADD, TRAF2, cIAP1, cIAP2, linear ubiquitin chain assembly complex (LUBAC) and RIP1, which leads to the highly ubiquitination of RIP1 resulting in NF-κb activation to promote gene transcription for inflammation and cell survival. Under conditions that RIP1 is deubiquitinated, TNF-α then triggers two programmed cell death: apoptosis or necroptosis. Deubiquitinated RIP1 forms a complex with FADD, FLIP and caspase-8 to trigger caspase-8 activation and subsequent apoptosis. Apoptosis generally blocks the necroptosis by caspase-8-mediated cleavage of RIP1 and RIP3 to inactivate RIP1 and RIP3. Upon caspase inhibition, RIP1 and RIP3 interact with each other through their RHIM domain and further recruit MLKL to trigger necroptosis [15, 16]. As alcohol consumption increases levels of TNF-α in the liver, therefore, it is not surprising that inflammation, apoptosis and necrosis can all be associated with the pathologies in both experimental and human ALD.

In the present study, we found that alcohol treatment increased the protein levels of RIP3 but not RIP1 in mouse livers, primary cultured mouse and human hepatocytes as well as alcoholic human livers. Interestingly, we found that Gao-binge alcohol treatment did not affect the hepatic mRNA levels of RIP3 but decreased hepatic mRNA levels of RIP1 in mouse livers. While it remains to be studied in the future how alcohol differentially regulates the transcription of RIP1 and RIP3 in the liver, these data clearly suggest that alcohol regulates RIP3 at the posttranslational but not at the transcriptional level. Indeed, we found that Gao-binge alcohol treatment decreased the protein levels of proteasome subunit PSMA2, PSMC1 and hepatic proteasome activity, which imply that impaired proteasomal degradation of RIP3 may be one underlying mechanism for RIP3 accumulation after alcohol exposure. The finding that pharmacological and genetic inhibition of proteasome in mouse livers increased RIP3 protein levels may further support this notion. It is known that acetaldehyde, one of the ethanol metabolites, is highly reactive and can modify protein structure and form protein adducts. It remains to be determined whether acetaldehyde may modify RIP3 structure and affects RIP3 protein stability and turnover. It should be noted that the basal hepatic expression levels of both RIP1 and RIP3 are quite low compared to the spleen and thymus in mice, suggesting that RIP1 and RIP3 may have critical roles in regulating immune response at the normal physiological conditions. However, under stress conditions such as the exposure to alcohol, RIP3 levels can be elevated due to the impaired proteasomal mediated turnover of RIP3 and may in turn trigger RIP3-mediated necroptosis.

In the present study, we found that blocking the kinase activity of RIP1 by 7-Nec1 did not affect Gao-binge alcohol treatment-induced cell death but significantly suppressed alcohol-induced expression of inflammatory genes, hepatic neutrophil infiltration and nuclear p65 translocation. These data suggest that the kinase activity of RIP1 is important for alcohol-induced inflammation but may be dispensable for alcohol-induced RIP3-mediated necroptosis. It should be noted that RIP1 protein levels already decreased in Gao-binge alcohol-treated mouse livers and in human ALD. However, this decrease is likely regulated at its transcriptional level since Gao-binge alcohol treatment decreased hepatic mRNA levels of RIP1. RIP1 can also be regulated by proteasome at the posttranslational level since blocking proteasome activity by a proteasome inhibitor increased both RIP1 and RIP3 protein levels. The decreased mRNA levels of RIP1 but not RIP3 after Gao-binge alcohol treatment may explain why only RIP3 protein levels were increased in hepatocytes after alcohol exposure despite of the alcohol-induced inhibition of hepatic proteasome functions. It should be noted that RIP3 KO mice already had increased basal hepatic inflammation compared with WT mice although hepatic inflammation was not different between WT and RIP3 KO mice after Gao-binge treatment. It is likely that the increased basal inflammation in RIP3 KO mice may also activate adaptive responses in RIP3 KO mice that cause RIP3 KO mice to be less sensitive to Gao-binge alcohol treatment. Nevertheless, our data suggest that RIP1 and RIP3 may differentially regulate different processes of alcohol-induced liver pathogenesis. RIP3 seems to be more important for mediating alcohol-induced necroptosis but RIP1 in particular its kinase activity seems to be more important for alcohol-induced inflammation.

In addition to ALD, increasing evidence indicates that RIP1-RIP3-mediated necroptosis occurs in many pathologically and physiologically relevant conditions. For instance, RIP1-RIP3-mediated necroptosis has been implicated in ischemic brain injury [43], ischemic-reperfusion-induced myocardial injury [44], kidney injury [45], pancreatitis [12], skin inflammation [46] and immune response against certain viral infections [12, 47]. In addition, we and others also recently reported that RIP3 and MLKL3 are important in acetaminophen-induced necrosis in mouse liver [48, 49]. A recent study reported that neither RIP3 nor MLKL was important but RIP1 was critical in acetaminophen-induced liver injury [50]. While different mouse strain or housing environment may be partially accountable for these contradicting observations, these data also highlighted the possible differential roles of RIP1 vs RIP3 in regulating different form of cell death and other cellular processes such as inflammation. For ALD, perhaps one of the intriguing puzzling questions is how both apoptosis and necroptosis are associated with the pathogenesis of ALD because apoptosis would normally suppress necroptosis by caspase-mediated cleavage of RIP3. It is possible that different cell death modes could predominate in different stages of ALD pathogenesis. For instance, apoptosis may occur in early ALD, such as in steatosis, whereas necroptosis may occur in late stages of ALD, such as in acute alcoholic hepatitis. These possibilities need to be further studied in the future. Moreover, the roles of several other RIP1 and RIP3 downstream players such as MLKL or PGAM5 in ALD also need to be further studied. Nevertheless, the possible switch between apoptosis and necroptosis raises concerns on the current ongoing clinical trial using a pan-caspase inhibitor for ALD [51]. Therefore, combination of small molecules that inhibit caspase and RIP1 as well as RIP3 may be a better approach for treating ALD than targeting either only apoptosis or necroptosis. Due to the differential role of RIP1 and RIP3 in regulating multiple cellular processes that are associated with ALD such as apoptosis, necroptosis and inflammation, it would also be better to target both RIP1 and RIP3 simultaneously.

## MATERIALS AND METHODS

### Reagents

Antibodies used in this study were β-actin (#A5441) from Sigma-Aldrich, Cleaved Caspase 3 (#9661L) and RIP1 (#3493) from Cell Signaling, RIP3 (#2283) from ProSci, CYP2E1 (#Ab19140), p65 (#sc-372) from Santa Cruz Biotechnology, PSMA2 (#Ab109525) and PSMB5 (#Ab3330) from Abcam, PSMC1(#A303-821A) from Bethyl, anti-lymphocyte antigen B superfamily (Ly6B, #MCA771G) from AbD Serotec, MCL-1 (#600-401-394) from Rockland. Horseradish peroxidase—conjugated secondary antibodies were from Jackson ImmunoResearch Laboratory. Ethanol was from Pharmaco, RIP1 Inhibitor 7-Cl-O-Nec-1 (7-Nec1, 504297) from Calbiochem and all other chemicals were from Sigma, Invitrogen or Calbiochem.

### Animals

Wild type (WT) male C57Bl/6J mice (Jackson Laboratories, Bar Harbor, ME) were used in this study. RIP3 KO mice were generously provided by Dr. Vishva Dixit (Genentech) as we described previously [22]. Liver-specific PSMC1 (RPT2) KO mice and matched control mice were described previously [23]. All mice were housed in cages (5 mice per cage) receiving a 12-hour light/dark cycle. All procedures were approved by the Institutional Animal Care and Use Committee of the University of Kansas Medical Center.

### Mouse Gao-binge alcohol treatment

Gao-binge alcohol treatment was performed as previously described [2, 24, 25], which was designed to mimic alcoholic liver injury in patients that had chronic alcohol abuse and binge drinking history. Briefly, male WT and RIP3 KO mice were fed ad libitum Lieber-DeCarli liquid (Bioserv # F1258SP) control diet for the first 5 days to adapt to the liquid diet. Then they sustained on a 5% (vol/vol) ethanol diet for the following 10 days. The 5% (vol/vol) ethanol diet provided 35.5% of total calories in the diet (10 days, 35.5%). Food consumption was recorded daily and weight was recorded every other days. The pair-fed mice received an isocaloric nonalcoholic control diet. On morning of the final day, mice were treated with 5 g/kg ethanol (Ethanol diet+ethanol, ED+E) or a 45% (w/v) maltose solution (Control diet+maltose, CD+M) via gavage. For some experiments, on the final day, one dose of 7-Nec-1 (5 mg/kg dissolved in 4% methyl-β-cyclodextrin in saline, i.p.) or vehicle (4% methyl-β-cyclodextrin in saline, i.p.) was given before a binge of ethanol (5 g/kg). Mice were euthanized 8 hours after the gavage, and blood samples and liver tissues were collected. Livers were extracted and weighed, and some portions of the liver were fixed in formalin for histopathological analysis or frozen in liquid nitrogen for biochemical assays. Liver injury was assessed by measuring serum alanine aminotransferase (ALT) activity as we described previously [26]. For the proteasome inhibition experiments, mice were treated with DMSO or Bortezomib (1 mg/kg, i.p.) for 24 hours followed by a second booster treatment for another 6 hours. Mice were euthanized and liver tissues were collected. Total liver lysates were prepared using RIPA buffer (1% NP40, 0.5% sodium deoxycholate, 0.1% sodium dodecyl (lauryl) sulfate).

### Histology and immunohistochemistry

Paraffin-embedded liver sections were stained with hematoxylin and eosin (H&E) for pathological evaluation. Frozen liver sections were dyed with Oil Red O staining for measuring fat accumulation as we described previously [25]. The images were further quantified by ImageJ using pixel number as the quantitative measurement as described previously [27]. Briefly, the pictures were first converted from RGB to grayscale formats, a threshold was set according to the staining and the integrated density was measured. Data were presented as the fold of control. Immunostaining for NF-κB subunit p65, RIP3, cleaved caspase-3 and Ly6B positive neutrophils was performed The percentage of hepatocytes that stained positive with different staining intensity for p65 was quantified. TUNEL staining was performed as we described previously [28].

### Primary hepatocytes culture

Murine hepatocytes were isolated by a retrograde, non-recirculating perfusion of livers with 0.05% Collagenase Type IV (Sigma) as described previously [29]. Cells were cultured in William's medium E with 10% fetal bovine serum, but no other supplements, for 2 hours to allow for attachment. Human hepatocytes were isolated and cultured according to the methods described previously by Gramignoli, et.al [30]. All cells were maintained in a 37°C incubator with 5% CO_2_. All human liver specimens were obtained in accordance with the KUMC Human Subjects Committee approved protocol # 13513.

### Immunoblot analysis

Twenty micrograms of protein from each sample were separated by SDS-PAGE and transferred to PVDF membranes. The membranes were immunoblotted with primary antibodies followed by secondary horseradish peroxidase-conjugated antibodies. The membranes were developed with SuperSignal West Pico chemiluminescent substrate (Pierce). Densitometry analysis was performed using Un-Scan-It software and normalized with β-actin. All the densitometry data were presented as means±SEM.

### Analysis of proteasome and caspase-3 activity

Total liver lysate (10 μg) was used for proteasome activity analysis using Suc-LLVY-AMC substrate (Enzo) as we described previously [31]. Briefly, lysates were diluted to 10 μg each per 15 μL of RIPA buffer and added to a white 96-well flat bottom plate. AMC substrate (20 nM) was added to each well along with 100 μL of assay buffer (50 mM Tris pH 7.5, 25 mM KCL, 10 mM NaCl, and 1 mM MgCl_2_ diluted in dH_2_O). Proteasome activity was determined by measuring AMC release using an excitation/emission 380/460 test filter on a Tecan plate reader after 1 hour. Data were expressed as fold of the control. Caspse-3 activity was measured using Ac-DEVD-AFC fluorescent substrate (Enzo) with 20 μg of protein from mouse liver lysates after designated treatment as we previously described [29].

### Triglyceride (TG) analysis

Hepatic TG extraction was performed as described previously [32]. Frozen liver tissues (50—100 mg) were crushed into powder using a mortar and pestle. One mL of a chloroform-methanol mixture (2:1) was added to the liver powder and shaken at room temp for 1 hour. Then 200 μL of ddH_2_O was added to the sample, and the mixture was centrifuged for 5 minutes at 3000g. The lower lipid phase was collected and dried, and the pellet was re-dissolved in Tert-Butanol-Triton X-114-methanol mix (2:3). TG analysis was carried out using a commercial kit (Pointe Scientific, Inc. GPO-Triglyceride Reagent Set) following the manufacturer's instructions.

### Quantitative real-time polymerase chain reaction (qRT-PCR)

RNA was extracted from mouse liver using Trizol (Invitrogen) and reverse transcribed into cDNA by RevertAid reverse transcriptase (Fermentas). Real-time PCR was performed on a Bio-Rad CFX384™ real-time PCR detection system using iTaq™ Universal SYBR® Green Supermix (Bio-Rad). Expression of *β-actin, RIP1, RIP3, Ly6B.2, IL-6, MCP-1 and TNF-α* was quantified using qRT-PCR analysis. Primers sequences were as follows: *β-actin* forward: 5′-TGTTACCAACTGGGACGACA-3′ and *β-actin* reverse: 5′-GGGGTGTTGAAGGTCTCAAA-3′; *RIP1* forward: 5′-CCTGCTGGAGAAGACAGACC-3′ and *RIP1* reverse 5′-CATCATCTTCCCCTCTTCCA-3′; *RIP3* forward: 5′-ACACGGCACTCCTTGGTATC-3′ and *RIP3* reverse: 5′-CCGAACTGTGCTTGGTCATA-3′; *Ly6G* forward: 5′-TGCGTTGCTCTGGAGATAGA-3′ and *Ly6G* reverse: 5′-CAGAGTAGTGGGGCAGATGG-3′; *IL-6* forward: 5′-ACAACCACGGCCTTCCCTACTT-3′ and *IL-6* reverse: 5′-CATTTCCACGATTTCCCAGAGA-3′; *MCP-1* forward: 5′-CCAGCCTACTCATTGGGAT-3′ and *MCP-1* reverse: 5′-ACATCCTGTATCCACACGGCAG-3′; *TNF-α* forward: 5′-CGTCAGCCGATTTGCT ATCT-3′ and *TNF-α* reverse: 5′-CGGACTCCGCAAAGTCTAAG-3′;

### Statistical analysis

All experimental data were expressed as means ± SE and subjected to Student t-test or one-way ANOVA with Bonferroni post hoc test where appropriate. *p<0.05 was considered significant.

## Abbreviations

ALD: Alcoholic liver disease
ALT: Alanine aminotransferase
CD+M: Control diet+maltose
cIAPs: inhibitor of apoptosis proteins
CYP2E1: Cytochrome P450 2E1
ED+E: Ethanol diet+ethanol
FLIPs: FLICE-like inhibitory proteins
GSH: glutathione
H & E: heamatoxylin and eosin
IRF3: interferon regulator factor 3
KO: knockout
Ly6B: anti-lymphocyte antigen B superfamily
MCL-1: Myeloid Cell Leukemia 1
MLKL: mixed lineage kinase domain-like
7-Nec1: 7-Cl-O-Nec-1
PSMA2: proteasome subunit alpha type-2
PSMB5: proteasome subunit beta type-5
PSMC1: proteasome 26S subunit ATPase 1
RHIM: RIP homotypic interaction motif
RIP1: Receptor-interacting serine/threonine-protein kinase 1
RIP3: Receptor-interacting serine/threonine-protein kinase 3
TG: Triglycerides
TNF-α: tumor necrosis factor-α.
